# Local and global mortality experience: A novel hierarchical model for regional mortality risk

**DOI:** 10.1371/journal.pone.0312928

**Published:** 2026-02-17

**Authors:** Asmik Nalmpatian, Christian Heumann, Levent Alkaya, William Jackson

**Affiliations:** Department of Statistics, LMU Munich, Munich, Bavaria, Germany; Cairo University, EGYPT

## Abstract

Accurate mortality risk assessment is critical for decision-making in life insurance, healthcare, and public policy. Regional variability in mortality, driven by diverse local factors and inconsistent data availability, presents significant modeling challenges. This study introduces a novel hierarchical mortality risk model that integrates global and local data, enhancing regional mortality estimation across diverse regions. The proposed approach employs a two-stage process: first, a global Light Gradient Boosting Machine model is trained on globally shared features; second, region-specific models are developed to incorporate local characteristics. This framework outperforms both purely local models and standard imputation techniques, particularly in data-scarce regions, by leveraging global patterns to improve generalization. The model is computationally efficient, scalable, and robust in handling missing values, making it adaptable for other domains requiring integration of multi-regional data. This method enhances predictive accuracy across various regions and provides a more reliable approach for mortality risk estimation in data-scarce environments.

## 1 Introduction

Mortality risk assessment plays a crucial role in various sectors, including life insurance, healthcare, and public policy. Reliable estimates of mortality rates are essential for strategic planning, policy formulation, and ensuring the financial stability of life insurance systems. However, accurately estimating mortality risk presents an essential challenge due to the diverse and dynamic nature of regional data availability and factors that affect mortality rates.

Hierarchical models have been utilized in mortality studies to account for variations at different levels, including regional, individual and national. Originally developed in fields like education, sociology, and demography, these models have gained significant traction in public health and epidemiology. By generalizing the classical pooling of group estimates, hierarchical or multilevel models offer a flexible framework for analyzing mortality data [1]. This flexibility allows researchers to better understand and interpret the complex factors influencing mortality rates across different populations.

Existing models in hierarchical mortality modeling include Bayesian approaches, generalized linear models, and machine learning (ML) techniques. Bayesian hierarchical models estimate mortality rates by incorporating prior distributions to handle uncertainty [2]. Generalized linear models, including multilevel Poisson regression, have been applied to mortality data to account for overdispersion and hierarchical structure [3]. Although the existing literature predominantly employs random effects for both methodologies, our approach diverges by sequentially processing the residuals. Recent studies have also explored ML methods such as random forests and gradient boosting for COVID-19 mortality modeling [4]. In general, the application of ML to mortality modeling raises important ethical and policy considerations, particularly with respect to fairness, transparency and potential biases embedded in training data [5,6]. While these topics are highly relevant for operational deployment, especially in settings such as life insurance pricing or public health planning, they are beyond the scope of this study. Our focus lies in benchmarking predictive models on a consistent dataset to evaluate methodological performance, rather than auditing societal impacts or implementing fairness-aware adjustments. Nevertheless, we recognize the importance of incorporating fairness constraints in future work where operational decisions may be affected.

Studies have highlighted the importance of balancing global patterns with local specifics in mortality modeling to ensure both generalizability and relevance [7,8]. However, the availability of mortality data varies widely across regions, posing challenges for model accuracy and reliability [9]. Poisson regression is commonly used for modeling count data, including mortality rates [10], whereas Light Gradient Boosting Machine (LightGBM) has been recognized for its efficiency and accuracy in handling large datasets, making it suitable for hierarchical mortality modeling [11]. We focus on gradient boosting due to its strong performance in high-dimensional, structured data and its ability to capture complex nonlinear interactions. While alternative ensemble methods exist, they offer limited additional insight relative to the methodological contribution of the hierarchical two-step framework.

Existing mortality models often struggle to balance global trends and local variations, leading to models that either overgeneralize or fail to capture region-specific nuances. Furthermore, inconsistent and sparse data availability across regions intensifies these challenges, reducing the reliability of predictions, especially in data-scarce environments [9]. Current approaches often suffer from overdispersion [12] or are computationally inefficient when handling large datasets or missing data [13]. These limitations underscore the need for a more flexible and scalable solution.

To address these challenges, this study introduces a novel hierarchical mortality modeling approach that integrates both global and local data. By using a two-stage process, our model first captures global patterns through a LightGBM model with a Poisson regression objective and then refines these predictions with region-specific models that account for local characteristics. While the first step includes shared variables that apply to all countries, such as age and gender, the country-specific models capture unique regional characteristics by incorporating additional region-specific factors, such as lifestyle habits and environmental conditions. This method markedly improves predictive performance, particularly in data-sparse regions, by leveraging global insights while remaining adaptable to the unique conditions of each region. Additionally, the model is computationally efficient, scalable, and capable of handling missing values, making it superior to traditional pooling methods. It is important to note that this study focuses explicitly on modeling mortality risk within the insured population, not the general public. Due to the effects of underwriting and self-selection, policyholders often exhibit lower average mortality than the general population [14,15]. Our goal is therefore not to generalize to national mortality rates, but to support more accurate estimation within life insurance portfolios. Beyond mortality risk estimation, this hierarchical modeling framework is applicable to other domains requiring multi-regional data integration, such as public health planning, epidemiological forecasting, and financial risk assessments. Its ability to generalize well across different regions makes it particularly valuable in scenarios where data sparsity or inconsistency is a common obstacle.

The structure of this paper is as follows: Sect 2 provides a brief overview of our database and Sect 3 presents our proposed methodology in detail. Sect 4 examines the effectiveness of our methodology by presenting and discussing the results. Finally, Sect 5 concludes by summarizing the main findings and suggesting research and industry perspectives.

## 2 Database

Data for the study was collected in a pseudonymised form from eight different operating units of a global primary insurance company, each representing a distinct country. Data privacy regulations prohibit the disclosure of these countries’ names, keeping the focus on the technical aspects of the model evaluation and comparison, rather than on potential privacy breaches. The chosen organizations were based on two key factors: having relevant data available of high quality and representing diverse geographic regions. Although individual country names are anonymized and represented by numerical identifiers due to contractual confidentiality with the data provider, the dataset spans multiple aggregated regions: four countries from Western Europe, one from Central and Eastern Europe, and three from Latin America. This regional diversity ensures substantial variation in demographic, economic, and insurance portfolio characteristics. The study’s objective is not to interpret country-specific mortality levels, but to evaluate methodological performance across heterogeneous regional contexts under controlled anonymization. To align local insurance mortality with broader national trends, we incorporate overall population mortality rates from the Human Mortality Database (HMD) [16]. These help bridge the gap between general population and insured portfolio data where needed. Nonetheless, the emphasis of this study remains on benchmarking methodological performance across different modeling strategies, rather than interpreting regional mortality variation. This international coverage provides diverse mortality experiences and helps assess the model’s robustness across different regional contexts [9].

The dataset includes policy data that remained active during this period, even if initially issued before the earliest year studied. In total, the dataset encompasses nearly 10 million life-years of exposure and close to 10,000 recorded insurance claims (=deaths).

The data underwent analysis in an aggregated form, grouped into *N* = 16.689.304 unique combinations of feature values. Specifically, the feature set *X*_*i*,*j*_, where group *i* ranges from 1 to *N* and *j* ranges from 1 to 8 - representing the eight countries, consists of a total of 26 features. Among these features, 9 are global, and up to 17 are local features, encompassing information about policyholders, insurance policies, and claims. The distinction between global and local features is based on their cross-country availability and actuarial relevance. Global features include universally recorded variables, while local features encompass country-specific attributes. Due to contractual confidentiality agreements, we cannot disclose raw feature names. However, the feature set can be described abstractly. It comprises standard actuarial and insurance portfolio variables, including demographic attributes (e.g., age and gender), policy characteristics (e.g., insured amount, premium structure, policy duration), portfolio exposure indicators (e.g., number of active policies), temporal information (calendar year), geographic classifications (e.g., region or state), underwriting and risk classification indicators (e.g., underwriting type, occupation class, premium loadings), distribution channel information, product characteristics, and contractual attributes such as premium type and policy currency. These feature categories are well established as key drivers of insured mortality and were selected based on actuarial domain expertise and consistent availability across countries.

Given these potential risk factors, our target is to model the number of deaths *D*_*i*,*j*_ in relation to the life years of risk exposure *E*_*i*,*j*_. The outcome variable is based on mortality events among insured individuals. As such, the dataset reflects a selected subpopulation shaped by underwriting criteria and insurance product design. This focus aligns with the study’s aim to develop predictive models for actuarial and risk management applications, rather than for population health surveillance. To facilitate model training and evaluation, an artificial variable was constructed before aggregating to create an 80-20 train-test split, ensuring that all unique combinations are adequately represented in both the training and test sets.

Table 1 summarizes deaths *D*_*j*_, exposure *E*_*j*_, number of feature combinations *N*_*j*_, and observed years per country *T*_*j*_. While sample sizes vary, including data-scarce countries is intentional, as the primary objective is to evaluate model robustness under heterogeneous data availability. Model performance is assessed within each country, ensuring that results are not confounded by differences in data volume across countries.

**Table 1:** Overview of death counts $D_{j}$, exposure in life years $E_{j}$, unique feature combination $N_{j}$, and observed years $T_{j}$ for each country *j.*

Country *j*	$D_{j}$	$E_{j}$	$N_{j}$	$T_{j}$
1	1699	1295299	1880792	2013–2020
2	1291	1686299	2190943	2010–2020
3	494	815795	1868691	2010–2020
4	1225	1347150	1572539	2017–2020
5	1816	1825901	4825792	2016–2020
6	2132	1548157	3852306	2016–2020
7	458	498560	207951	2017–2020
8	297	99473	290290	2015–2020
**Total**	9412	9116634	16689304	2010–2020

The discrepancy in feature sets and values across countries results in missing blocks. Missing values occur exclusively in local features due to structural differences in feature availability across countries, while global features are complete by design. Table 2 reports the percentage of missing values per feature and country. We do not observe systematic missingness affecting entire countries across all features; rather, missingness reflects realistic country-specific data availability. The two-step modeling framework explicitly accommodates this structure by isolating globally complete features in the first step and incorporating local features only where available. We have imputed the missing values based on feature type: categorical features receive “Missing” and metric features receive “–1”. This approach retains information from non-missing values and identifies missing values during interactions for local features. In contrast, global features are free from missing values due to the design of the data collection process.

**Table 2:** Percentage of missing values in each feature by country.

Country	F1	F2	F3	F4	F5	F6	F7	F8	F9	F10	F11	F12	F13	F14	F15	F16	F17	F18	F19	F20	F21	F22	F23	F24	F25	F26
1	0	0	0	0	0	0	0	0	0	0	0	33	0	72	0	0	0	0	0	0	0	72	72	72	72	0
2	0	0	0	0	0	0	0	0	0	0	0	4	0	0	0	0	0	0	0	0	48	48	48	48	48	48
3	0	0	0	0	0	0	0	0	0	0	0	0	0	0	0	33	33	0	0	0	0	33	5	6	0	0
4	0	0	0	0	0	0	0	0	0	0	0	28	0	0	0	28	0	28	0	0	0	28	0	28	28	28
5	0	0	0	0	0	0	0	0	0	0	0	4	0	0	0	0	0	0	0	0	0	72	0	100	100	0
6	0	0	0	0	0	0	0	0	0	0	0	37	0	0	0	62	0	0	0	0	62	58	56	0	62	0
7	0	0	0	0	0	0	0	0	0	0	0	0	0	0	8	2	8	0	6	0	8	8	8	8	8	8
8	0	0	0	0	0	0	0	0	0	0	0	0	0	6	0	6	0	0	0	6	6	0	0	6	6	6

## 3 Methodology

The foundation of our approach is rooted in the Cox Proportional Hazards model (Cox PH), a class of survival models in statistics that aligns with our objective of estimating mortality rates [17]. To simplify the complexity of Cox PH model calculations, we leveraged the connection between Cox PH and a Poisson Generalized Linear Model (GLM). Assuming piecewise constant hazard rates over time, the likelihood of the Cox PH model coincides with the likelihood of the Poisson GLM when we employ *log*(*E*_*i*,*j*_) as an offset parameter, as detailed by [18] who noted, “we do not assume [the Poisson model] is true, but simply use it as a device for deriving the likelihood”. Independent of [18,19] published a similar insight, emphasizing that the piece-wise proportional hazards model is equivalent to a specific Poisson regression model.

Our primary goal is to accurately evaluate mortality rates. We aim to estimate the conditional expectation of death counts, denoted as *D*_*i*,*j*_, given the available information summarized in the feature set *X*_*i*,*j*_ and the exposure in life years at risk *E*_*i*,*j*_. The feature set includes both numerical and categorical variables. Categorical features were encoded using target encoding, whereby each category is replaced by the mean of the target variable conditional on that category. To prevent target leakage, target encoding was performed within a k-fold cross-validation framework: for each fold, encoding statistics were computed exclusively on the training portion and applied to the validation fold. This approach is particularly effective for high-cardinality categorical features and avoids the dimensionality explosion associated with one-hot encoding. Assuming that $D_{i,j}\overset{\mathrm{ind}.}{\sim }\text{ Poisson}(\mu_{i,j}\;\cdot \;E_{i,j})$, the expectation according to the Poisson distributional assumption is:


$$𝔼[D_{i,j}|X_{i,j},E_{i,j}]=\mu_{i,j}\;\cdot \;E_{i,j}=\exp\left(X_{i,j}^{⊺}\beta_{j}\right)\;\cdot \;E_{i,j}$$

The Poisson log-likelihood is defined:


$$\left(\beta_{j}|X_{i,j},D_{i,j})=\sum_{i=1}^{N_{j}}(D_{i,j}\;\cdot \;\log({\hat{D}}_{i,j})-{\hat{D}}_{i,j})\right)$$

where *D*_*i*,*j*_ denotes the observed death counts, ${\hat{D}}_{i,j}={\hat{\mu }}_{i,j}\;\cdot \;E_{i,j}$ denotes the predicted death counts, and $\mu_{i,j}=\exp\left(X_{i,j}^{⊺}\beta_{j}\right)$ with $\beta_{j}$ as the parameter vector.

This formulation assumes that deaths follow a Poisson distribution. An advantage of simplifying the Cox PH model into a Poisson GLM is its adaptability to the ML realm, requiring optimization using Poisson log-likelihood and the ability to define an offset or observation weights. ML models, which generally do not assume specific (i.e. additive) relationships between features and targets, can leverage this flexibility:


$$𝔼[D_{i,j}|X_{i,j}]=\mu_{i,j}\;\cdot \;E_{i,j}=\exp(f(X_{i,j}))\;\cdot \;E_{i,j}$$

This transition from GLMs to ML models offers additional benefits, including integrated variable selection mechanisms and the ability to capture interactions without explicit specification.

To implement this approach, we employ the LightGBM algorithm [11], a popular ML technique based on boosting. LightGBM iteratively builds an ensemble of decision trees to model the relationship between features and the target variable, optimizing the model to minimize the negative log-likelihood of the Poisson distribution [20]. Trees are fit to residuals derived from the loss function, and the model is updated iteratively to minimize this loss. The prediction is formulated as a linear combination of the base learners:


$$\mu_{i,j}=\exp(f(X_{i,j}|\theta ))=\exp\left(\sum_{k=0}^{K}\theta_{k}\;\cdot \;u_{k}(X_{i,j})\right)$$

where $\theta_{k}$ is the weight of the *k*-th tree, and $u_{k}(X_{i,j})=\sum_{l\in V_{k}}b_{l}\;\cdot \;𝕀[X_{i,j}\in R_{l}]$ represents the tree associated with $V_{k}$ as set of leaves of the *k*-th tree, *b*_*l*_ as the predicted value in the *l*-th leaf, and *R*_*l*_ as the region defined by disjoint partitions of the training set associated with the *l*-th leaf [21]. LightGBM uses a leaf-wise growth strategy, splitting the leaf with the highest loss reduction first, and adopts a histogram-based algorithm to improve the efficiency and speed of building decision trees. This approach results in efficient and accurate models, particularly for datasets with complex or imbalanced relationships.

To distinguish between local and global features and ensure high accuracy in each country, we propose a Two-step model approach. Hierarchical (multi-level) models offer theoretical advantages because they allow partial pooling of information across groups. By borrowing strength across regions, a hierarchical model reduces the variance of local estimates while still permitting group-specific effects [1]. This tends to yield more accurate and stable mortality risk estimates in regions with sparse data compared to a single-step approach [13]. While LightGBM does not implement formal hierarchical shrinkage as in Bayesian mixed-effects models, the proposed two-step framework provides an operational approximation. The global model pools information across all countries, while local models learn residual country-specific deviations. This induces an implicit shrinkage effect, whereby countries with limited data remain closer to the global pattern, whereas data-rich countries exhibit stronger local adjustments. This approach involves two distinct modeling steps:

**Step 1: Global model**: The first model identifies global patterns and uses a training set that includes data from all countries, focusing solely on “global” factors. These global factors are those where data across countries is comparable, such as age. In contrast, factors like postal code, which lack comparability between regions, are excluded. The residuals $\frac{D_{i,j}}{{\hat{D}}_{i,j}^{\text{global}}}$ represent the deviation of the observed deaths from the expected deaths predicted by the first step,

**Step 2: Specialized Local model**: In the second step, we calculate one Local model per country, totaling eight Local models. Each Local model takes the output of the Global model and adjusts it to the specific circumstances of the respective country. The initial weights for this step are the expected deaths from the first step, ${\hat{D}}_{i}^{\text{global}}$. Specialized Local models use all global factors plus the country-specific local factors. The distinction of the feature set into global and local features is based on the availability of data across countries as well as domain-specific expert knowledge. Details on prediction calibration are provided in S3 Appendix.

**Fig 1:**
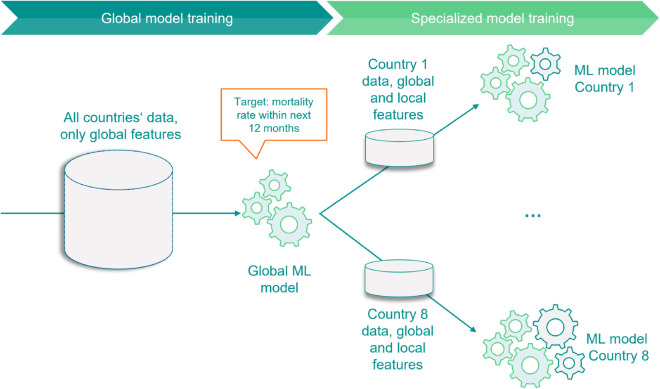
Qualitative illustration of the proposed methodology, with gearwheels representing the features.

This approach combines the estimates from both the global and specialized Local models as illustrated in Fig 1.

Mathematically, we can express the process of estimating death counts for a policy with given factors as follows:


$$𝔼[D_{i,j}|X_{i,j}]=\mu_{i,j}\;\cdot \;E_{i,j}=q(X_{i,j}^{\text{global}})\;\cdot \;h_{j}(X_{i,j}^{\text{all}})\;\cdot \;E_{i,j}$$

where *D*_*i*,*j*_ represents the expected number of deaths given a set of features *X*_*i*,*j*_ for group *i* and country *j*; q(·) represents the Global model’s prediction function; $h_{j}(\cdot )$ represents the Local model’s prediction function for country *j*; $X_{i,j}^{\text{global}}$ represents a set of factor values for group *i* and country *j*, containing only global factors; $X_{i,j}^{\text{all}}$ represents a set of factor values for country *j*, containing both global and local factors.

In technical terms, the predicted mortality rates from the first Global model are used to initialise the second specialized Local model. Accordingly, the model continues to work on the resulting residuals and iteratively optimises the second model - but now with the broader, localised data set. The final predicted number of deaths results from the multiplication of the predictions from the Global model (first step), the predictions from the specialised Local model (second step) and the exposure. The following derivation shows that the multiplication is justified by the nature of the boosting algorithm and the exponentiation by the log link of the Poisson distribution:


$$\mu_{i,j}=\exp\left(\sum_{k=1}^{K}\theta_{k}\;.u_{k}(X_{i,j})\right)\;=\prod_{k=1}^{K}\exp(\theta_{k}.u_{k}(X_{i,j}))\overset{g:=\exp(\theta .u(X))}{=}\underset{\mathrm{Global}\;\mathrm{model}=q(.)}{\underbrace{\prod_{k=1}^{P}g_{k}(X_{i,j})}}.\underset{\mathrm{Local}\;\mathrm{model}=h_{j}(.)}{\underbrace{\prod_{l=P+1}^{K}g_{l}(X_{i,j})}}$$

Splitting the modeling into two steps offers the advantage of cleanly separating effects into local and global categories. It also optimizes model performance for each market by tailoring the model to local patterns while allowing knowledge sharing across countries via the Global model. Additionally, when onboarding a new country, we can choose to retain the existing Global model and calculate a new Local model for this new country.

As the LightGBM software does not allow the inclusion of an offset, we utilize observed mortality rates as the target variable, thus the death counts are scaled by exposure $D_{i,j}/E_{i,j}$ and exposure *E*_*i*,*j*_ is used as weights, a method demonstrated to be mathematically equivalent in the Poisson case by [22]. The model validation strategy follows a structured procedure and explicitly accounts for the hierarchical data structure through cross-validation, sequential global-to-local model tuning, and country-specific out-of-sample evaluation. This ensures generalization across both countries and time. The dataset is split into training (80%) and test (20%) sets using a predefined fold variable. Hyperparameters are tuned via grouped 5-fold cross-validation stratified by country-year combinations on the training data using the Hyperopt library [23]. The global model is tuned first. Subsequently, out-of-sample predictions from the global model are used to train and tune the country-specific local models, ensuring that no information leakage occurs between modeling stages. To mitigate overfitting, we impose minimum thresholds on exposure and death counts, apply regularization through the global pooling step, and evaluate train-test performance gaps using RMSE, correlation, and Poisson likelihood. Additional automated checks include agreement plots by age and gender, smoothness assessments, and comparisons against standalone local models. These diagnostics ensure both statistical and actuarial plausibility Mechanisms we employ to control overfitting and ensure robust performance are detailed in S2 Appendix.

## 4 Benchmarking results

**Fig 2:**
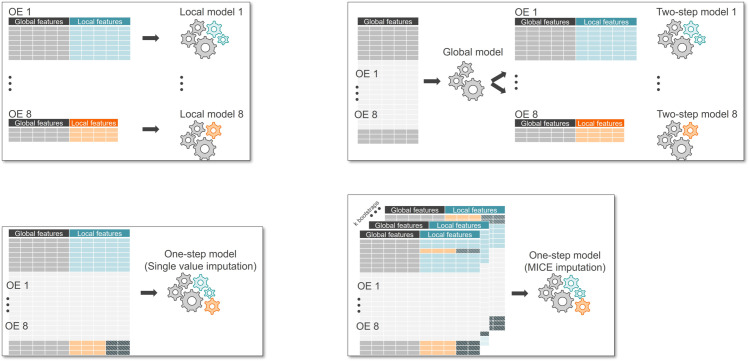
Comparison of the benchmarked models and their frameworks, with gearwheels representing the features. Grey stands for global features, blue and orange for local features specific to different countries, and patterned dark cells indicate missing values. A. Local model. B. Two-step model. C. One-step model with single value imputation. D. One-step model with MICE.

Our objective is to benchmark the proposed methodology against three other approaches using specific evaluation metrics. This aims to determine the predictive performance and computational efficiency of the proposed model compared to the alternatives. All these methods are based on the model specification proposed in the previous section, where death counts are estimated in relation to exposure using the ML model LightGBM, optimizing the Poisson log-likelihood assumption. Classical multilevel models were not included, as they are less suited to high-dimensional feature spaces with complex nonlinear interactions and structured missingness across countries. The proposed approach prioritizes scalability, flexibility, and predictive performance under realistic operational constraints common in insurance data. The differences among these methods are outlined below and illustrated in Fig 2:

*1. Local models for individual countries:* For each country, we take this country’s data and run the model separately. This is, of course, only applicable if we have enough claims and exposure available for a given country as a solid foundation for training. The information contained in the each other countries about certain features and their correlation patterns to mortality rates remain unseen for each model.

*2. Two-step approach:* As detailed in the previous section, this approach combines global features in the first step model, using common features across countries. In the second step, a Local model is trained to capture also each country’s specificities based on residuals from the first step.

*3. Global one-step with single value imputation:* All datasets from different countries are combined in this early data fusion technique. In cases where a local model cannot be trained due to small data size, the One-Step approach may be the only viable option, but it results in missing blocks that must be imputed. The Two-Step model offers a valuable alternative by providing flexibility: if a local feature is entirely missing, it can be dropped, similar to local models, while global features are retained based on global patterns. For partially missing local features, single value imputation is applied, and the researcher has the option to drop or keep the imputed feature for a specific country. We chose to retain all features that are not completely missing within a country to ensure no information is lost.

*4. Global one-step with bootstrapped multiple imputation:* Similar to the previous approach, this method involves early data fusion by combining datasets from all countries. In this case, we use Bootstrapped Multiple Imputation with Decision Tree as imputation technique for missing values that arise due to the synthetic dataset creation. The procedure is as follows:

- First draws k bootstrap samples from the combined dataset including missing values.
- Fit a classification or regression tree by recursive partitioning, variable by variable.
- After fitting a tree for the missing value based on the other values of the variable from the corresponding leaf, a value is randomly drawn.

This ensures that we can use it properly for multiple imputation, so that we are inducing some variation and not just the randomness in the leaf. The implementation was done in Python [24] with an adapted version of IterativeImputer [25], using 4 bootstrap samples and 2 imputations iterations each. We refer to [26] for further algorithm details. The number of iterations was determined based on a trial-and-error approach, as higher numbers had no significant impact on the final model results due to the dataset’s size. Based on each dataset resulting from the bootstrapped iteration, we trained the proposed model and finally pooled the eight predictions by averaging. The baseline is the pure local model trained separately per country. MICE is included as one of several benchmark approaches to represent a more sophisticated imputation strategy, allowing comparison against simpler imputation and the proposed two-step framework.

**Evaluation criteria:** To evaluate our proposed methodology, we place a strong emphasis on two critical dimensions: predictive accuracy and computational efficiency.

To gauge the predictive performance of our models, we employ two essential metrics: Root Mean Square Error (RMSE) for both in-sample and out-of-sample assessments. For a given country *j* it is calculated as follows:


$$RMSE_{j}=\sqrt{\sum_{i=1}^{N_{j}}({\hat{D}}_{i,j}-D_{i,j}{)}^{2}}$$

Additionally, we utilize the Poisson log-likelihood, which serves a dual role as a loss function and evaluation metric:


$$l_{j}=\sum_{i=1}^{N_{j}}\left(D_{i,j}\;\cdot \;\log({\hat{D}}_{i,j})-{\hat{D}}_{i,j}\right)$$

In the equations, ${\hat{D}}_{i,j}={\hat{\mu }}_{i,j}\;\cdot \;E_{i,j}$ represents the predicted, while *D*_*i*,*j*_ the observed death counts. The in-sample metrics allow us to examine how well the model fits the training data. On the other hand, the out-of-sample metrics serves as a litmus test for the model’s ability to generalise to new, unseen data.

A higher log-likelihood and lower RMSE signify a closer fit between the model and the data, indicating superior performance. Conversely, a lower log-likelihood and higher RMSE are indicative of a less suitable model for the given data.

We consider runtime, memory usage, and storage requirements to evaluate the computational efficiency of our models, aiming for lower values to enhance their practical utility. These criteria offer a comprehensive assessment of our models’ performance in estimating mortality rates and pricing life insurance.

**Outcomes:** This section details the benchmarking process for all four models, focusing on key metrics for performance and efficiency assessment. We evaluated the models using multiple metrics, including train and test RMSE and log-likelihood. Although RMSE is reported, log-likelihood is more reliable due to the distributional assumptions of the data. Additionally, we assessed computational efficiency through run time (seconds), memory consumption (megabytes), and storage space of the model object (kilobytes).

In Tables 3 and 4 we present the results exemplarily for country 5 and 7, and in S2 Appendix an overview of all countries as well as the cross-country results. Each table provides an insight into the performance of the four benchmarked models, highlighting their strengths and weaknesses in various aspects. For ease of interpretation, we have used colour coding in dark grey to identify the best model within each row, based on the respective metric. The comparison is based on original values, before rounding for readability reasons.

**Table 3:** Performance evaluation for country 5.

Metric	Local model	Two-step model	One-step model (Single Value)	One-step model (MICE)
RMSE (Train)	$1.990\times 10^{-2}$	bg=gray!60$1.979\times 10^{-2}$	$2.009\times 10^{-2}$	$2.126\times 10^{-2}$
RMSE (Test)	$1.709\times 10^{-2}$	bg=gray!60$1.706\times 10^{-2}$	$1.709\times 10^{-2}$	$1.811\times 10^{-2}$
Log Likelihood (Train)	$-1.110\times 10^{4}$	bg=gray!60$-1.069\times 10^{4}$	$-1.295\times 10^{4}$	$-1.315\times 10^{4}$
Log Likelihood (Test)	$-3.429\times 10^{3}$	bg=gray!60$-3.399\times 10^{3}$	$-3.938\times 10^{3}$	$-3.998\times 10^{3}$
Runtime (Sec)	$1.370\times 10^{4}$	bg=gray!60$3.970\times 10^{2}$	–	–
Memory (MB)	$2.998\times 10^{3}$	bg=gray!60$1.663\times 10^{2}$	–	–
Storage (KB)	$2.174\times 10^{6}$	bg=gray!60$2.162\times 10^{6}$	–	–

**Table 4:** Performance evaluation for country 7.

Metric	Local model	Two-step model	One-step model (Single Value)	One-step model (MICE)
RMSE (Train)	$5.358\times 10^{-2}$	bg=gray!60$5.061\times 10^{-2}$	$5.736\times 10^{-2}$	$5.847\times 10^{-2}$
RMSE (Test)	bg=gray!60$3.542\times 10^{-2}$	$3.604\times 10^{-2}$	$3.983\times 10^{-2}$	$3.714\times 10^{-2}$
Log Likelihood (Train)	$-1.821\times 10^{3}$	bg=gray!60$-1.469\times 10^{3}$	$-2.439\times 10^{3}$	$-2.682\times 10^{3}$
Log Likelihood (Test)	$-5.615\times 10^{2}$	bg=gray!60$-5.529\times 10^{2}$	$-5.682\times 10^{2}$	$-5.693\times 10^{2}$
Runtime (Sec)	$9.144\times 10^{2}$	gray!80y$1.518\times 10^{1}$	–	–
Memory (MB)	$3.041\times 10^{2}$	bg=gray!60$1.983\times 10^{2}$	–	–
Storage (KB)	$9.816\times 10^{4}$	bg=gray!60$9.376\times 10^{4}$	–	–

Our Two-step modeling approach demonstrates the best predictive performance for nearly all countries, as evidenced by our comprehensive evaluation. This method outperforms Local models in most cases and shows significant advantages over the MICE method. Detailed results can be found in the tables and figures, highlighting the effectiveness of our approach.

The Two-step model shows the most substantial improvements for smaller countries (e.g., countries 7 and 8), compared to larger countries (e.g., countries 4 and 5). This is particularly evident in the test log-likelihood improvements from Local models to the Two-step model. By leveraging a Global model in the first step, we protect local specifics while enhancing the generalization capability, especially for smaller datasets.

Our research compares also one-step models, including single value imputation and MICE, with the proposed two-step approach. The findings consistently show that one-step models underperform and demand substantial computational resources. Specifically, MICE exhibits inferior performance for country-specific results. In terms of storage, single value imputation slightly outperforms the proposed model, if considered both steps. However, the one-step approaches require full retraining when new data becomes available, which can impact results for other countries.

When considering computational efficiency, encompassing aspects like runtime and memory consumption, the two-step approach stands out as the preferred choice. It’s important to emphasise that the performance of Local models is closely linked to the availability and quality of data within a given country. While this study has the privilege of using high-quality data with rich claims and exposures, this may not be the case for every country or data source. In such cases, the two-step approach with its cross-country learning capabilities provides a distinct advantage, as we can use the insights gained from the Global model to retrain the second step of the process. Although the main analysis is conducted under a Poisson distributional assumption, we also experimented with a Negative Binomial specification to assess robustness to overdispersion [10,19]. The relative performance of the benchmarking approaches remained stable, with the two-step model consistently outperforming the others. However, the absolute fit metrics were uniformly lower across all models under the Negative Binomial assumption. Given these results, and to ensure comparability across techniques, we retain the Poisson model in the main analysis. Full results from the Negative Binomial experiments are provided in S4 Appendix.

Overall, our proposed two-step hierarchical modeling approach achieves superior predictive performance for nearly all countries, outperforming Local models and the MICE method, with log-likelihood proving to be a more reliable measure than RMSE due to the distributional assumptions of the data generation process. The Two-step model significantly enhances generalization for smaller countries, such as countries 7 and 8, by leveraging a Global model in the first step, which protects local specifics and improves performance even stronger compared to larger countries like countries 4 and 5. From a computational perspective, the two-step hierarchical framework adds a layer of complexity by requiring both a global model and multiple local refinements. However, this structure benefits from efficiency: the global model’s outputs are reused for initializing local models, and local model training is parallelizable across regions. Empirically, we observed a moderate increase in total training time, around 15–20% compared to a single-step model, primarily due to repeated evaluations and retraining phases. Importantly, the global model needs to be trained only once and can be applied to additional countries without retraining, making the approach scalable and efficient for expanding to new regions. Given the consistent performance improvements in predictive accuracy, we view this trade-off as justifiable in practical actuarial applications where accuracy and interpretability are paramount. Although this study focuses on within-dataset benchmarking, the generalizability of the hierarchical modeling framework has been further explored in a dedicated follow-up study [27]. That study applies the global pretrained model to the United Kingdom, where no internal portfolio data was available, and adapts it using a similarity-weighted synthetic dataset constructed from external indicators and HMD data. The transfer learning approach achieves strong agreement with UK mortality benchmarks, including the Continuous Mortality Investigation (CMI) tables, thus demonstrating the framework’s applicability in data-poor environments.

## 5 Summary and outlook

This study introduces a novel two-stage hierarchical mortality model that integrates global and local data to improve regional mortality risk estimation, particularly in data-scarce regions. The model leverages a LightGBM in the first stage to capture global patterns, followed by country-specific refinements in the second stage. This approach demonstrated superior predictive accuracy compared to traditional methods and effectively addressed challenges related to missing data, scalability, and overgeneralization, offering a robust solution for mortality risk modeling across diverse regions.

The two-stage hierarchical modeling approach not only enhances predictive performance but also offers practical benefits in fields such as life insurance pricing, risk assessment, and public health planning. By generating more accurate mortality risk estimates, particularly in regions with limited local data, the model supports better-informed decision-making in industries that rely on precise risk evaluations. Its scalability and computational efficiency make it especially valuable in large-scale, multi-regional contexts. Accurate regional mortality predictions have important policy implications: they can inform public health resource allocation, insurance premium setting, and pension planning [6].

Our model also stands out for its computational efficiency, excelling in runtime, memory usage, and storage requirements, particularly when the first-stage global model is excluded. This efficiency is advantageous when scaling to new countries, as only the second step requires retraining, leaving existing predictions unaffected. The reduced model size speeds up training times while maintaining high performance, making it suitable for applications where rapid training is essential. Additionally, the model provides an efficient solution for handling missing data, outperforming other methods like single-value imputation or MICE, particularly when working with small datasets where local data alone is insufficient, and the pre-learned knowledge of a larger model becomes critical.

Despite its strong performance across multiple regions, the model’s effectiveness depends on the availability and quality of data. In regions with low or inconsistent data quality, future research could explore more advanced imputation techniques or alternative methods for managing missing data. Further work could also investigate optimizing computational efficiency for even larger datasets or extending the model’s applicability to domains such as epidemiological forecasting, financial risk modeling, or public health surveillance. Integrating techniques like deep learning could enhance performance for more complex datasets, though this may compromise its interpretability. Finally, we emphasize that ML models can inadvertently propagate biases present in the training data. Future work should incorporate fairness-aware ML techniques [28] to ensure that mortality estimates remain equitable across demographic groups and do not reinforce existing disparities [29].

The flexibility and robustness of the proposed hierarchical model open up new possibilities for accurate risk estimation, particularly in data-scarce environments. As industries continue to rely on precise mortality estimates for strategic decision-making, this approach sets the foundation for more reliable, scalable, and adaptable models capable of addressing the complexities of regional variability without compromising performance.

## Supporting information

S1 Appendix Rest of country-specific results. (PDF)

S2 Appendix Hyperparameter optimization. (PDF)

S3 Appendix Evaluation of prediction calibration. (PDF)

S4 Appendix Sensitivity analysis using alternative distributions. (PDF)
