# Supplementary material for: Local and global mortality experience: A novel hierarchical model for regional mortality risk
Source: PLoS One. 2026 Feb 17;21(2):e0312928. doi: 10.1371/journal.pone.0312928 (PMC12912697; doi:10.1371/journal.pone.0312928)
Supplement: S1 Appendix — (PDF) [file pone.0312928.s001.pdf]

S1 Appendix. Rest of country-specific results

Table 1. Cross-country evaluation of computational efficiency

Metric	Local model	Two-step model (Step 1 & 2)	One-step model (Single Value)	One-step model (MICE)
Runtime (Sec)	4.586×10^4	$6.122 \times 10^3 + 8.064 \times 10^2$	4.696×10^4	5.119×10^5
Memory (MB)	1.382×10^4	$5.562 \times 10^2 + 4.909 \times 10^3$	1.069×10^4	2.004×10^5
Storage (KB)	7.903×10^6	$8.816 \times 10^6 + 7.837 \times 10^6$	6.669×10^6	5.785×10^7

Table 2. Performance evaluation for country 1

Metric	Local model	Two-step model	One-step model (Single Value)	One-step model (MICE)
RMSE (Train)	2.509×10^{-2}	2.506×10^{-2}	2.539×10^{-2}	2.614×10^{-2}
RMSE (Test)	2.181×10^{-2}	2.180×10^{-2}	2.210×10^{-2}	2.259×10^{-2}
Log Likelihood (Train)	-6.575×10^3	-6.538×10^3	-7.927×10^3	-7.916×10^3
Log Likelihood (Test)	-2.071×10^3	-2.066×10^3	-2.409×10^3	-2.459×10^3
Runtime (Sec)	3.565×10^3	3.832×10^1	-	-
Memory (MB)	1.301×10^3	7.233×10^1	-	-
Storage (KB)	8.420×10^5	8.371×10^5	-	-

Table 3. Performance evaluation for country 2

Metric	Local model	Two-step model	One-step model (Single Value)	One-step model (MICE)
RMSE (Train)	2.631×10^{-2}	2.628×10^{-2}	2.853×10^{-2}	2.855×10^{-2}
RMSE (Test)	1.872×10^{-2}	1.872×10^{-2}	1.920×10^{-2}	1.941×10^{-2}
Log Likelihood (Train)	-7.877×10^3	-7.673×10^3	-8.017×10^3	-8.201×10^3
Log Likelihood (Test)	-2.152×10^3	-2.151×10^3	-2.542×10^3	-2.537×10^3
Runtime (Sec)	2.759×10^3	0.988×10^2	-	-
Memory (MB)	1.580×10^3	5.512×10^2	-	-
Storage (KB)	9.751×10^5	9.688×10^5	-	-

Table 4. Performance evaluation for country 3

Metric	Local model	Two-step model	One-step model (Single Value)	One-step model (MICE)
RMSE (Train)	1.674×10^{-2}	1.673×10^{-2}	1.764×10^{-2}	1.783×10^{-2}
RMSE (Test)	1.329×10^{-2}	1.328×10^{-2}	1.328×10^{-2}	1.331×10^{-2}
Log Likelihood (Train)	-3.314×10^3	-3.119×10^3	-3.471×10^3	-3.479×10^3
Log Likelihood (Test)	-9.515×10^2	-9.506×10^2	-1.109×10^3	-1.210×10^3
Runtime (Sec)	2.387×10^3	9.700×10^1	-	-
Memory (MB)	1.324×10^3	9.292×10^2	-	-
Storage (KB)	8.326×10^5	8.279×10^5	-	-

Table 5. Performance evaluation for country 4

Metric	Local model	Two-step model	One-step model (Single Value)	One-step model (MICE)
RMSE (Train)	2.999×10^{-2}	2.219×10^{-2}	3.183×10^{-2}	3.183×10^{-2}
RMSE (Test)	2.219×10^{-2}	2.998×10^{-2}	3.307×10^{-2}	3.307×10^{-2}
Log Likelihood (Train)	-6.842×10^{3}	-6.730×10^{3}	-1.166×10^{4}	-1.167×10^{4}
Log Likelihood (Test)	-1.907×10^{3}	-1.900×10^{3}	-2.275×10^{3}	-2.275×10^{3}
Runtime (Sec)	1.772×10^{3}	1.401×10^{1}	-	-
Memory (MB)	1.084×10^{3}	1.074×10^{3}	-	-
Storage (KB)	7.004×10^{5}	6.954×10^{5}	-	-

Table 6. Performance evaluation for country 6

Metric	Local model	Two-step model	One-step model (Single Value)	One-step model (MICE)
RMSE (Train)	2.404×10^{-2}	2.396×10^{-2}	2.731×10^{-2}	2.846×10^{-2}
RMSE (Test)	2.129×10^{-2}	2.128×10^{-2}	2.565×10^{-2}	2.648×10^{-2}
Log Likelihood (Train)	-1.081×10^{4}	-1.060×10^{4}	-1.191×10^{4}	-1.285×10^{4}
Log Likelihood (Test)	-3.194×10^{3}	-3.186×10^{3}	-3.492×10^{3}	-3.621×10^{3}
Runtime (Sec)	1.093×10^{4}	3.421×10^{3}	-	-
Memory (MB)	2.666×10^{3}	1.331×10^{3}	-	-
Storage (KB)	1.735×10^{6}	1.724×10^{6}	-	-

Table 7. Performance evaluation for country 8

Metric	Local model	Two-step model	One-step model (Single Value)	One-step model (MICE)
RMSE (Train)	3.283×10^{-2}	3.230×10^{-2}	3.304×10^{-2}	3.514×10^{-2}
RMSE (Test)	2.909×10^{-2}	2.906×10^{-2}	2.907×10^{-2}	2.955×10^{-2}
Log Likelihood (Train)	-1.247×10^{3}	-1.546×10^{3}	-1.730×10^{3}	-1.845×10^{3}
Log Likelihood (Test)	-5.275×10^{2}	-5.211×10^{2}	-6.318×10^{2}	-6.398×10^{2}
Runtime (Sec)	5.235×10^{2}	4.571×10^{1}	-	-
Memory (MB)	2.641×10^{2}	8.588×10^{1}	-	-
Storage (KB)	1.334×10^{5}	1.303×10^{5}	-	-
