# Supplementary material for: Local and global mortality experience: A novel hierarchical model for regional mortality risk
Source: PLoS One. 2026 Feb 17;21(2):e0312928. doi: 10.1371/journal.pone.0312928 (PMC12912697; doi:10.1371/journal.pone.0312928)
Supplement: S2 Appendix — (PDF) [file pone.0312928.s002.pdf]

S2 Appendix. Hyperparameter optimization

For the hyperparameter optimization we used Hyperopt, a Python library that uses the Tree Parzen Estimator (TPE) algorithm. TPE is an efficient method leveraging a probabilistic model to guide the search for optimal hyperparameters. TPE workflow can be briefly characterized as follows:

Initialisation: TPE starts by randomly sampling a few hyperparameter combinations to create an initial set of observations. These initial combinations serve as a starting point for the optimisation process.

Probabilistic Modelling: TPE uses probabilistic models to capture the relationship between hyperparameter values and the performance metric (e.g., loss or accuracy). Specifically, it models the probability that a configuration will produce better results.

Exploitation and exploration: Based on the probabilistic models, TPE tries to balance exploration and exploitation. It aims to understand the correlation between hyperparameter values and performance, emphasising configurations that are likely to lead to better results. This process is similar to the gradient descent algorithm, but instead of searching for the gradient of the loss function, it focuses on the probability distribution of the hyperparameters.

Updating the model: As TPE collects more observations and evaluates additional configurations, it updates its probabilistic models. The algorithm iteratively learns and refines its models to make more informed decisions.

By iteratively balancing exploration and exploitation, TPE efficiently navigates the hyperparameter space, eventually converging on an optimal set of hyperparameters for a given ML model. For a deeper understanding of the TPE algorithm and practical application we refer to [2] and [1]. The optimized hyperparameters of the discussed models are represented in Table 1 (For the sake of clarity and simplicity, MICE has been excluded).

Table 1. Optimal Hyperparameters for Local, Two-step and One-step models

Country	n_estimators	subsample	colsample_bytree	num_leaves	min_child_samples	learning_rate
**Local models**						
1	1806	0.8247	0.7842	51	76	0.01
2	1452	0.7526	0.5450	6	78	0.01
3	627	0.6610	0.5325	4	49	0.01
4	1118	0.5877	0.5378	4	89	0.01
5	1875	0.9231	0.6487	50	13	0.01
6	1223	0.8147	0.5253	15	41	0.01
7	1197	0.9384	0.8898	51	5	0.01
8	1990	0.7399	0.6098	5	80	0.01
**Two-step model**						
1st step	441	0.7833	0.8400	27	734	0.1
2nd step - 1	1488	0.6711	0.9983	9	644	0.01
2nd step - 2	322	0.9983	0.8304	98	992	0.01
2nd step - 3	1580	0.5496	0.6262	97	43	0.01
2nd step - 4	895	0.8998	0.8485	44	666	0.01
2nd step - 5	1786	0.9210	0.8615	94	294	0.01
2nd step - 6	1993	0.7889	0.8863	18	735	0.01
2nd step - 7	82	0.7024	0.6998	22	421	0.01
2nd step - 8	1538	0.7188	0.6480	78	166	0.01
**One-step model (Single Value)**						
–	1355	0.5807	0.8553	17	296	0.05

References

1. Bergstra J, Yamins D, Cox D. Making a science of model search: Hyperparameter optimization in hundreds of dimensions for vision architectures. In: Proceedings of the International Conference on Machine Learning. 2013. p. 115–123.

2. Bergstra J, Bardenet R, Bengio Y, Kégl B. Algorithms for hyper-parameter optimization. Adv Neural Inf Process Syst. 2011;24.
