# Supplementary material for: Local and global mortality experience: A novel hierarchical model for regional mortality risk
Source: PLoS One. 2026 Feb 17;21(2):e0312928. doi: 10.1371/journal.pone.0312928 (PMC12912697; doi:10.1371/journal.pone.0312928)
Supplement: S3 Appendix — (PDF) [file pone.0312928.s003.pdf]

S3 Appendix. Evaluation of prediction calibration

The balance property is a critical consideration in the context of statistical modeling. It signifies that a well-calibrated model should exhibit no deviations between the mean of observed and predicted values. This property is satisfied for any generalized linear model (GLM) within the exponential dispersion family (EDF) as long as the canonical link function is employed ([1], [3]). However, deviations can arise when the chosen link function departs from the canonical link. In such cases, it's essential to address this issue. [2] provides a relatively straightforward approach to correct for balance property failures, ensuring that the model remains well-calibrated.

To mitigate these issues and achieve calibration, we leverage Gradient Boosting advantages, which allows us to optimize the model with a Poisson loss function, implicitly using a log-link function. This strategic choice corrects any balance property problems and results in a well-calibrated model. Table 1 below illustrates that our models, in general, exhibit either no deviations or very minimal differences between observed and predicted death counts. Calibration is more meaningful when applied to training data, where models are meticulously adapted and fine-tuned. Any minor deviations observed in the test set are typically of negligible significance. For the Two-step model, the final mean predictions are computed by multiplying the means from the first and second steps, ensuring robustness in our approach.

Table 1. Predicted vs. observed avg. death counts (Train/Test)

Country		1	2	3	4	5	6	7	8
Local models									
Predicted Mean (Train)		0.0013	0.0008	0.0006	0.0009	0.001	0.0014	0.0009	0.003
Observed Mean (Train)		0.0013	0.0008	0.0006	0.0009	0.001	0.0014	0.0009	0.003
Predicted Mean (Test)		0.0013	0.0008	0.0006	0.0009	0.001	0.0014	0.0008	0.003
Observed Mean (Test)		0.0012	0.0008	0.0006	0.0009	0.0009	0.0013	0.0008	0.0029
Two-step model	1st step	2nd step							
Predicted Mean (Train)	0.001	1.0302	0.9826	0.9791	0.9813	0.9833	0.9885	1.0031	1.056
Observed Mean (Train)	0.001	1.0337	0.9908	0.98	0.9855	0.9958	0.9892	1.0049	1.0639
Predicted Mean (Test)	0.001	1.0295	0.984	0.9805	0.9826	0.955	0.9952	1.003	1.0566
Observed Mean (Test)	0.001	0.9687	1.0172	0.9351	0.9218	0.9101	0.9094	0.9027	1.0156
One-step model (Single-Value)									
Predicted Mean (Train)		0.0013	0.0008	0.0006	0.0009	0.001	0.0014	0.0009	0.003
Observed Mean (Train)		0.0013	0.0008	0.0006	0.0009	0.001	0.0014	0.0009	0.003
Predicted Mean (Test)		0.0013	0.0008	0.0006	0.0009	0.001	0.0014	0.0009	0.0029
Observed Mean (Test)		0.0012	0.0008	0.0006	0.0009	0.0009	0.0013	0.0008	0.0029

References

1. Noll A, Salzmann R, Wüthrich MV. Case study: French motor third-party liability claims. SSRN. 2020.

2. Wüthrich MV. Bias regularization in neural network models for general insurance pricing. Eur Actuar J. 2020;10(1):179–202.

3. Wüthrich MV. The balance property in neural network modelling. Stat Theory Relat Fields. 2022;6(1):1–9.
