# Supplementary material for: Local and global mortality experience: A novel hierarchical model for regional mortality risk
Source: PLoS One. 2026 Feb 17;21(2):e0312928. doi: 10.1371/journal.pone.0312928 (PMC12912697; doi:10.1371/journal.pone.0312928)
Supplement: S4 Appendix — (PDF) [file pone.0312928.s004.pdf]

S4 Appendix. Sensitivity analysis using alternative distributions

To assess robustness against overdispersion, we complemented our Poisson-based modeling framework with an alternative specification assuming a Negative Binomial (NB) distribution. This extension allows the variance of death counts to exceed the mean, capturing additional heterogeneity in mortality outcomes.

Let $D_{i,j} \in \mathbb{N}_0$ denote the observed number of deaths and $E_{i,j}$ the exposure in life-years for group i in country j, regardless of global or local models. Define the predicted mortality rate as $\hat{\mu}_{i,j} = \exp(f_\theta(X_{i,j}))$, where f_θ is the LightGBM prediction function, and the predicted number of deaths as $\hat{D}_{i,j} = \hat{\mu}_{i,j} \cdot E_{i,j}$. we now assume a Negative Binomial distribution of the form:

$$D_{i,j} \overset{\text{ind.}}{\sim} \text{NegBin}(\hat{\mu}_{i,j} \cdot E_{i,j},\ r),$$

where the mean is $\mathbb{E}[D_{i,j}] = \hat{\mu}_{i,j} \cdot E_{i,j}$ and the variance is:

$$\text{Var}(D_{i,j}) = \hat{\mu}_{i,j} \cdot E_{i,j} + \frac{(\hat{\mu}_{i,j} \cdot E_{i,j})^2}{r}.$$

The parameter $r > 0$ governs the degree of overdispersion. The Negative Binomial log-likelihood, omitting constants not depending on the model parameters, is then given by:

$$\ell_{NB}(\hat{\mu}_{i,j} \mid D_{i,j}, E_{i,j}; r) = \sum_{i=1}^{N_j} \left[D_{i,j} \cdot \log\left(\frac{\hat{D}_{i,j}}{r + \hat{D}_{i,j}} \right) + r \cdot \log\left(\frac{r}{r + \hat{D}_{i,j}} \right) \right].$$

We implement this loss function as a custom objective in LightGBM. Since the software does not support joint estimation of the dispersion parameter, we fix r externally and treat it as a hyperparameter. Observed mortality rates $\mu_{i,j} = D_{i,j}/E_{i,j}$ are used as the regression targets, while exposures $E_{i,j}$ serve as sample weights. This setup is mathematically equivalent to maximizing the log-likelihood shown above, as demonstrated in the Poisson case.

The estimation follows the gradient boosting paradigm: at each iteration, LightGBM fits a regression tree to the first-order gradients of the loss and uses the second-order derivatives (Hessians) for curvature correction in the Newton update. For the NB loss, gradients and Hessians are derived analytically from the log-likelihood expression. The boosting model iteratively minimizes the negative log-likelihood, estimating $\mu_{i,j}$ while holding r fixed.

While the relative ranking of model performance remained unchanged under the NB specification, we observed uniformly lower fit metrics across all methods compared to their Poisson counterparts. The results, summarized in Tables 1 and 2, show that although all models exhibit approximately 10% lower fit on average compared to their Poisson-based counterparts, the relative performance ranking remains stable. Specifically, the two-step model continues to outperform the benchmark models, reaffirming that the observed methodological gains are not sensitive to the choice of distribution. These findings support our decision to retain the Poisson assumption in the main analysis to ensure a consistent benchmarking framework across all methods. Two key reasons likely explain this behavior: Data has little or no overdispersion and

the Poisson assumption may already be adequate due to underwriting effects, which tend to reduce variance. The NB objective introduces additional curvature and dependency on r, which can lead to less stable convergence and slightly noisier tree splits during boosting. Further research could leverage alternative estimation techniques for the overdispersion parameter.

These findings reinforce the robustness of our methodological conclusions while supporting the use of Poisson likelihood for benchmark comparability.

Table 1. Performance evaluation for the Two-step model with Negative Binomial distributional assumption

Country	1	2	3	4	5	6	7	8
Metric	Country 1	Country 2	Country 3	Country 4	Country 5	Country 6	Country 7	Country 8
RMSE (Train)	2.559×10^{-2}	2.684×10^{-2}	1.707×10^{-2}	3.059×10^{-2}	2.030×10^{-2}	2.452×10^{-2}	5.465×10^{-2}	3.349×10^{-2}
RMSE (Test)	2.225×10^{-2}	1.909×10^{-2}	1.356×10^{-2}	2.263×10^{-2}	1.743×10^{-2}	2.172×10^{-2}	3.613×10^{-2}	2.967×10^{-2}
Log Likelihood (Train)	-6.707×10^{3}	-8.035×10^{3}	-3.380×10^{3}	-6.979×10^{3}	-1.132×10^{4}	-1.103×10^{4}	-1.857×10^{3}	-1.272×10^{3}
Log Likelihood (Test)	-2.112×10^{3}	-2.195×10^{3}	-9.705×10^{2}	-1.945×10^{3}	-3.498×10^{3}	-3.258×10^{3}	-5.727×10^{2}	-5.380×10^{2}
Runtime (Sec)	3.636×10^{3}	2.814×10^{3}	2.435×10^{3}	1.807×10^{3}	1.397×10^{4}	1.115×10^{4}	9.327×10^{2}	5.340×10^{2}
Memory (MB)	1.327×10^{3}	1.612×10^{3}	1.350×10^{3}	1.106×10^{3}	3.058×10^{3}	2.719×10^{3}	3.102×10^{2}	2.694×10^{2}
Storage (KB)	8.588×10^{5}	9.946×10^{5}	8.492×10^{5}	7.144×10^{5}	2.217×10^{6}	1.770×10^{6}	1.002×10^{5}	1.361×10^{5}

Table 2. Performance evaluation for the Local model with Negative Binomial distributional assumption

Country	1	2	3	4	5	6	7	8
RMSE (Train)	2.634×10^{-2}	2.792×10^{-2}	1.757×10^{-2}	3.149×10^{-2}	2.090×10^{-2}	2.524×10^{-2}	5.626×10^{-2}	3.447×10^{-2}
RMSE (Test)	2.290×10^{-2}	1.965×10^{-2}	1.395×10^{-2}	2.330×10^{-2}	1.794×10^{-2}	2.235×10^{-2}	3.719×10^{-2}	3.054×10^{-2}
Log Likelihood (Train)	-6.904×10^{3}	-8.271×10^{3}	-3.480×10^{3}	-7.184×10^{3}	-1.165×10^{4}	-1.135×10^{4}	-1.912×10^{3}	-1.309×10^{3}
Log Likelihood (Test)	-2.174×10^{3}	-2.259×10^{3}	-9.990×10^{2}	-2.002×10^{3}	-3.600×10^{3}	-3.354×10^{3}	-5.896×10^{2}	-5.539×10^{2}
Runtime (Sec)	3.743×10^{3}	2.897×10^{3}	2.506×10^{3}	1.861×10^{3}	1.439×10^{4}	1.147×10^{4}	9.601×10^{2}	5.497×10^{2}
Memory (MB)	1.366×10^{3}	1.659×10^{3}	1.390×10^{3}	1.138×10^{3}	3.148×10^{3}	2.799×10^{3}	3.193×10^{2}	2.773×10^{2}
Storage (KB)	8.841×10^{5}	1.023×10^{6}	8.742×10^{5}	7.354×10^{5}	2.283×10^{6}	1.822×10^{6}	1.030×10^{5}	1.401×10^{5}
